# Complete mitochondrial genome of the Japanese bumblebee, *Bombus hypocrita sapporensis* (Insecta: Hymenoptera: Apidae)

**DOI:** 10.1080/23802359.2016.1155423

**Published:** 2016-03-28

**Authors:** Jun-ichi Takahashi, Mana Nishimoto, Takeshi Wakamiya, Moe Takahashi, Takuya Kiyoshi, Koji Tsuchida, Tetsuro Nomura

**Affiliations:** a Department of Life sciences, Kyoto Sangyo University, Kyoto, Japan;; b Faculty of Applied Biological Sciences, Gifu University, Gifu, Japan;; c Department of Zoology, National Museum of Nature and Science, Tokyo, Japan

**Keywords:** Asian bumblebee, *Bombus hypocrite*, genetic distance, identification

## Abstract

In the present study, we describe the complete mitochondrial genome of the bumblebee, *Bombus hypocrita sapporensis* from the Rebun Island, in Hokkaido, Japan. The mitochondrial genome of *B. hypocrita sapporensis* includes a circular molecule of 15 700 bp. It contains 13 protein-coding genes, 22 tRNA genes, two rDNA genes and an A + T-rich control region. All protein-coding genes are initiated by ATA, ATG, and ATT codons and are terminated by the typical stop codon TAA or T, except for *ND4L*, which ends with TA. All tRNA genes typically form a cloverleaf secondary structure.

The Asian orange-tailed bumblebee, *Bombus hypocrita*, is a potential pollinator of greenhouse tomatoes that are distributed in Far East Asia. Mitochondrial DNA sequences of *B. hypocrita sapporensis* in South Korea have been analyzed except for the complete sequences of two genes (12S rDNA and tRNA-Gln) (Hong et al. 2008). This is the first study on the successful determination of the complete sequence of mitochondrial DNA of *B. hypocrita sapporensis* in Japan (accession number: AP017370).

An adult worker was collected from the Rebun Island in Hokkaido, Japan (Specimen is stored in the National Museum of Nature and Science, Japan accession number: NSMT-I-HYM 73172). Genomic DNA isolated from the worker was sequenced using Illumina’s HiSeq platform. The resultant reads were assembled and analyzed using the MITOS web server (Bernt et al. 2013, Germany) and GENETYX v.10 (GENETYX Corporation, Japan). Phylogenetic analysis was performed using the MEGA7 (Tamura et al. 2013) based on the nucleotide sequences of the 13 protein-coding genes.

The *B. hypocrita sapporensis* mitochondrial genome forms a 15 700 bp closed loop. This mitochondrial genome represents a typical hymenopteran mitochondrial genome and matches with the *B. ignites* genome, in which it comprises 13 protein-coding genes, 22 putative tRNA genes, two rDNA genes and an A + T-rich control region (Cha et al. 2007; Du et al. 2015). The average AT content of the *B. hypocrita sapporensis* mitochondrial genome was 85.1%. Similar to the bumblebee mitochondrial genomes, the heavy strand (H-strand) encodes nine protein-coding genes and 14 tRNA genes, and the light strand (L-strand) encodes four protein-coding genes, eight tRNAs and two rDNA genes. The *ATP8* and *ATP6* genes shared 17 nucleotides, the *ATP6* and *COIII* genes shared one nucleotide, and the *ND6* and *Cytb* shared 11 nucleotides. Six protein-coding genes of the *B. hypocrita* mitochondrial genome started with ATA, the *ATP6*, *COIII*, *ND4* and *Cytb* genes with ATG, and the *ATP8* and *ND5* genes with ATT, all of which have been commonly found in the South Korean *B. hypocrita sapporensis* mitochondrial genome (Hong et al. 2008). The stop codon of each of these protein-coding genes was either TAA or T except for *ND4L* gene with TA, similar to the case in other bumblebees. All of the tRNA genes typically possessed cloverleaf secondary structures.

Nucleotide substitution rate between the *B. hypocrita sapporensis* mitochondrial genomes of Japan and South Korea was 99.0% (15 310/15 468). This corresponds well with the genetic distances generally between the *Apis cerana* mitochondrial genomes of Japan and China (Tan et al. 2011; Takahashi et al. 2016). Phylogenetic analysis was performed by applying 13 mitochondrial protein-coding genes with 12 closely related taxa (Figure 1). Classification of bumblebees is difficult because of the similarity between the female morphological traits of different species. In this study, phylogenetic analysis based on the mitochondrial DNA aided in the classification of the species of bumblebee. Complete sequence of the mitochondrial DNA in bumblebees is an effective information for species classification.

**Figure 1.**
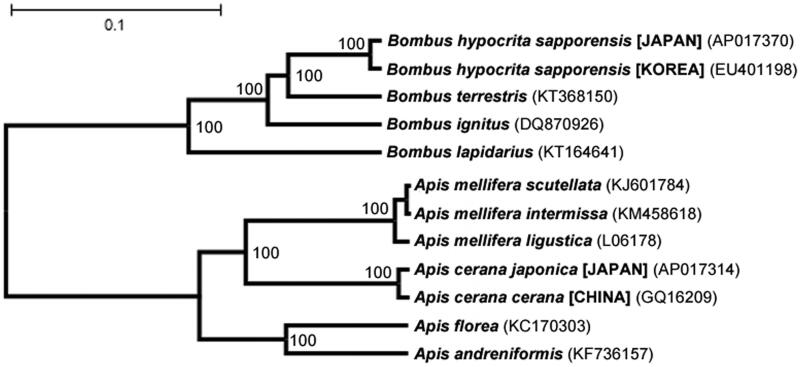
Phylogenetic relationships between the genera *Bombus* and *Apis* based on the mitochondrial genome nucleotide sequence of 13 protein-coding genes. Lengths on each node indicate the genetic distances supported in the analysis of Kimura two parameter model. Numbers beside nodes are percentage of 1000 bootstrap values. Alphanumeric symbols in parentheses indicate the GenBank accession numbers.
